# Impact of the COVID-19 pandemic on children with and without affective dysregulation and their families

**DOI:** 10.1007/s00787-022-02106-3

**Published:** 2022-11-16

**Authors:** A.-K. Treier, V. Holas, A. Görtz-Dorten, F. Frenk, C. Goldbeck, K. Mücke, C. Hanisch, A. Ritschel, V. Roessner, J. Rothe, U. Ravens-Sieberer, A. Kaman, T. Banaschewski, D. Brandeis, P.-M. Aggensteiner, M. Kölch, A. Daunke, M. Döpfner, for the ADOPT Consortium

**Affiliations:** 1 Department of Child and Adolescent Psychiatry, Psychosomatics and Psychotherapy, Faculty of Medicine and University Hospital Cologne, University Cologne, Cologne, Germany; 2 School for Child and Adolescent Cognitive Behavior Therapy (AKiP), Faculty of Medicine and University Hospital Cologne, University of Cologne, Cologne, Germany; 3 Faculty of Human Sciences, University of Cologne, Cologne, Germany; 4 Department of Child and Adolescent Psychiatry and Psychotherapy, TU Dresden, Dresden, Germany; 5 Department of Child and Adolescent Psychiatry, Psychotherapy, and Psychosomatics & Research Unit Child Public Health, University Medical Center Hamburg-Eppendorf, Hamburg, Germany; 6 Department of Child and Adolescent Psychiatry and Psychotherapy, Central Institute of Mental Health, Medical Faculty Mannheim, University of Heidelberg, Mannheim, Germany; 7 Department of Child and Adolescent Psychiatry and Psychotherapy, Psychiatric Hospital, University of Zürich, Zurich, Switzerland; 8 Zürich Center for Integrative Human Physiology, University of Zürich, Zurich, Switzerland; 9 Neuroscience Center Zürich, University and ETH Zürich, Zurich, Switzerland; 10 Department of Child and Adolescent Psychiatry, Neurology, Psychosomatics, and Psychotherapy, University Medical Center Rostock, Rostock, Germany; 11 Department of Child and Adolescent Psychiatry/Psychotherapy, University of Ulm, Ulm, Germany

**Keywords:** COVID-19, Children and adolescents, Stress, Risk factors, Affective dysregulation

## Abstract

Analyzing COVID-19-related stress in children with affective dysregulation (AD) seems especially interesting, as these children typically show heightened reactivity to potential stressors and an increased use of maladaptive emotion regulation strategies. Children in out-of-home care often show similar characteristics to those with AD. Since COVID-19 has led to interruptions in psychotherapy for children with mental health problems and to potentially reduced resources to implement treatment strategies in daily life in families or in out-of-home care, these children might show a particularly strong increase in stress levels. In this study, 512 families of children without AD and 269 families of children with AD reported on COVID-19-related stress. The sample comprised screened community, clinical, and out-of-home care samples. Sociodemographic factors, characteristics of child and caregiver before the pandemic, and perceived change in external conditions due to the pandemic were examined as potential risk or protective factors. Interestingly, only small differences emerged between families of children with and without AD or between subsamples: families of children with AD and families in out-of-home care were affected slightly more, but in few domains. Improvements and deteriorations in treatment-related effects balanced each other out. Overall, the most stable and strongest risk factor for COVID-19-related stress was perceived negative change in external conditions—particularly family conditions and leisure options. Additionally, caregiver characteristics emerged as risk factors across most models. Actions to support families during the pandemic should, therefore, facilitate external conditions and focus on caregiver characteristic to reduce familial COVID-19-related stress. *Trial registration: *German Clinical Trials Register (DRKS), ADOPT Online: DRKS00014963 registered 27 June 2018, ADOPT Treatment: DRKS00013317 registered 27 September 2018, ADOPT Institution: DRKS00014581 registered 04 July 2018.

**Supplementary Information:**

The online version contains supplementary material available at 10.1007/s00787-022-02106-3.

## Introduction

Following the first reported cases of COVID-19 in China in December 2019, the virus spread rapidly across the world (1). On March 11th 2020, the WHO officially declared the COVID-19 outbreak a pandemic, and shortly thereafter, Europe became a pandemic epicenter, accounting for over 40% of all confirmed cases worldwide. Extensive actions including school closures, contact restrictions, working from home, and public locality closures were established in Germany to reduce the risk of transmission of the COVID-19 virus—the first so-called ‘lockdown’. After some of these restrictions were lifted in April and May 2020, prevalence rates began to rise again in late summer, leading to further restrictions in October and a second full lockdown in December 2020.

As a result of these measures, families were faced with persistent changes to their daily life. In accordance with these changes, empirical data suggest a negative effect of the COVID-19 crisis on the mental health of children and parents/caregivers/guardians (henceforth ‘caregivers’). Studies applying various operationalizations of stress found that 15–71% of children [2–4] and 30–75% of caregivers reported elevated levels of stress due to the pandemic [2, 3, 5]. Moreover, many studies have demonstrated increased levels of mental health problems as a consequence of COVID-19 [3, 5–7]. However, besides these negative pandemic-related changes, the crisis may have also alleviated some strain for families [8]. For instance, homeschooling may have limited negative feedback from teachers or peers and might have provided more time for family activities or discovering new hobbies [9]. While there is little research to date on pandemic-related stress reduction, some studies have reported improvements in at least one mental health domain [4, 10, 11].

Generally, stress is experienced when objective and subjective external or internal demands exceed an individual’s resources [12]. One facet of an individual’s resources is emotion regulation abilities, which play a crucial role in the perception of stress. Indeed, prospective studies have identified low emotion regulation abilities of the child or caregivers as an important risk factor for elevated stress levels following COVID-19 measures [2, 6, 13]. In addition to emotion regulation abilities, individuals also differ in the threshold and intensity of their emotional reactivity to potentially stressful stimuli and increased or reduced reactivity is related to psychopathology [12].

Children with affective dysregulation (AD) show a pattern of both an elevated use of maladaptive emotion regulation strategies and heightened emotional reactivity [14, 15]. Typically, AD comprises severe temper outbursts, anger, and unpredictable mood swings that are inappropriate for the child’s developmental age [16]. The same pattern of an elevated use of maladaptive emotion regulation strategies and heightened emotional reactivity is often found in children in out-of-home care who have been maltreated by their parents [17, 18]. Such children have been exposed to unresponsive parenting and have modeled their parents’ heightened emotional reactivity and negative emotion regulation [17]. Due to the role of emotion regulation strategies and reactivity in the perception of stress, and in view of the increased external demands due to COVID-19, both children with AD and children in out-of-home care might show a particularly strong increase in stress levels in response to the pandemic. Therefore, the first aim of this study was to determine possible differences in COVID-19-related stress between children with and without AD and between children in out-of-home care and children in adoptive or biological families according to child and caregiver report.

Considering the potentially increased stress levels due to COVID-19, access to psychotherapeutic care might be particularly essential [19]. Due to the pandemic-related restrictions, however, psychotherapeutic care has been disrupted or limited for most families, and at least some sessions have been delivered online rather than face-to-face. Additionally, families might have reduced resources to implement treatment strategies in daily life. These changes in psychotherapeutic care might have been especially harmful for vulnerable children with pre-existing disorders such as AD. On the other hand, online therapy might have reduced some of the barriers to attending psychotherapeutic treatment such as time and costs for travel or childcare for siblings [20]. Thus, the second aim of the study was to determine potential treatment-related changes due to COVID-19 in children with AD.

To be able to adequately support families in times of COVID-19, it is necessary to identify risk and protective factors for COVID-19-related stress. The most commonly reported risk factors in studies focusing on COVID-19-related stress are sociodemographic variables such as higher or lower age of child, higher parental age, female or male gender of child, female gender of parent, higher educational level of child, lower socioeconomic status, and migration background (e.g. [2–4, 13, 21]). Some studies have focused on pre-existing mental disorders as potential risk factors. Studies examining children with and without pre-existing mental disorders (e.g. depression, attention-deficit hyperactivity disorder) reported differences in specific domains, although COVID-19-related changes were relatively comparable overall (e.g. [6, 10]). Moreover, research has indicated an influence of familial risk and protective factors, such as parental mental health and parenting behavior, on the child’s stress levels [22]. Finally, several studies have focused on external conditions due to COVID-19—that is, actual changes in the environment such as lockdown, job loss, or changes in the family setting [13, 23, 24]. Thus, the third aim of this study was to identify potential risk and protective factors for COVID-19-related stress in children with and without AD and their caregivers, including sociodemographic variables, pre-existing child and caregiver psychopathological symptoms, parenting, familial protective factors, and COVID-19-related changes in external conditions such as family or working conditions.

## Methods

### Participants

The present sample comprised subsamples of the research consortium ADOPT (Affective Dysregulation—Optimizing Prevention and Treatment), which analyzes the effects of stepped care interventions in children with AD [14].

#### Pre-pandemic recruitment

Before the pandemic, three samples with AD (AD group, *n* = 487) were recruited based on high scores on a caregiver-rated screening for AD in the ADOPT project (DADYS-Screen; ≥ 90th percentile; [25]: one screened community sample and one clinical sample (with adoptive or biological caregivers) and one out-of-home care sample. Additionally, three samples without AD (No AD group, *n* = 821) were recruited: one screened community sample with typical or subclinical scores (> 10th and < 90th percentile; No AD_11-89_) and one screened community sample with very low scores (≤ 10th percentile, No AD_0-10_)—both with either adoptive or biological caregivers—and one out-of-home care sample with typical, subclinical, or very low scores (< 90th percentile; No AD_0-89_). The screened community samples were recruited through residents’ registration offices in four German cities. The clinical sample was recruited in six child psychiatric outpatient units in Germany and the out-of-home care samples were recruited through out-of-home care institutions and foster families. For inclusion in the ADOPT trials following the screening, patients had to fulfill the following criteria: age between eight and 12 years, intelligence quotient above 80, no mental disorder that could explain the symptoms of AD (e.g. autism spectrum disorder), and no current behavioral therapy for the treatment of AD. Families in the AD sample who participated in the subsequent trials were randomized to a treatment or a treatment as usual (TAU) condition.

#### Pre-pandemic data collection

Data on potential risk and protective factors including sociodemographic characteristics, child characteristics, and caregiver characteristics were taken from the first in-depth assessment following the screening questionnaire, in which clinical, caregiver, and child report were used. In the No AD_11-89_ group, only caregiver report was assessed. Predictors were assessed between 2 November 2018 and 22 December 2020, although 96.2% of these data were collected by 28 February 2020, before the implementation of the COVID-19 measures. All data were collected either online via the REDcap electronic data capture tool hosted at the Clinical Trials Centre Cologne or offline in pen-and-paper format.

#### COVID-19 data collection

All families who participated in the intensive assessment following the screening questionnaire were asked to participate in the present study on COVID-19-related stress (*n* = 1308). The response rate for the assessment of COVID-19-related stress was 59.7% (*n* = 781). The patient flow and the composition of the present sample are depicted in the flowchart in figure S1. The COVID-19-related data were collected between 28 May 2020 and 22 December 2020, although 97% of the data were collected by 31 July 2020. The interval between the assessment of the predictors and the assessment of COVID-19-related stress ranged from 0 to 28 months, with a mean of 14 months (*SD* = 3.99).

### Measures

#### COVID-19-related stress

The *Corona Child Stress Scale* (CCSS; [26], see table S1) captures pandemic-related changes in relationships with family and friends, changes in child internalizing and externalizing symptoms, changes in school, working or family conditions, and changes in treatment according to caregiver report (CCSS-P: 14 items) and child report (CCSS-C: nine items). Some of the items were based on the CRISIS questionnaire [27], translated and adapted for our research purposes and sample. The items were rated on a 5-point Likert scale ranging from -2 (= much less stress) to 2 (much more stress). We developed two main scales: a total stress scale for all families (CCSS-P-Stress: 11 items, CCSS-C-Stress: seven items) and a treatment-related caregiver rating scale in the subgroup receiving therapy (CCSS-P-Treat: three items). In addition, we calculated the CCSS-P-Stress-internal (seven items) subscale, consisting of those items of the caregiver-rated total stress scale focusing on internal factors—that is, the perceived stress following changes in external conditions. Mean item scores were calculated for each subscale. In the current sample, internal consistency of the subscales was sufficient to good, ranging from α = 0.74 to α = 0.82.

#### Predictors of COVID-19-related stress

##### Sociodemographic characteristics

We considered gender of child, age and country of birth of child and caregivers, native language (German, other than German) and migration background of child (native, first generation, second generation) in line with PISA [28], school type (primary, secondary), school grade, special educational needs (yes, no), number of biological parents, single parent status of primary caregiver (yes, no), educational and occupational status of caregivers based on Lampert et al. [29], and family adversity based on the Family Adversity Index (cf. [30]).

##### Child characteristics

AD symptoms were assessed using the Diagnostic Tool for Affective Dysregulation in Children (DADYS; [31]). The DADYS captures emotional lability, emotion regulation, negative emotional reactions, and negative mood in clinical interviews for caregivers (13 items) and children (10 items) and in questionnaires for caregivers (36 items) and children (26 items). As a second measure of AD, we assessed the subscales anxious/depressed (13 items), attention problems (10 items), and aggressive behavior (18 items) of the Dysregulation Profile [32] from the German version of the caregiver-rated Child Behavior Checklist (CBCL/6-18R); [33]. Mental disorders were assessed using the Diagnostic System for Mental Disorders in Children and Adolescents according to ICD-10 and DSM-5 (DISYPS-III); [34]. From the DISYPS-III, we deployed the therapist-rated diagnostic screening checklists for internalizing symptoms (19 items) and externalizing symptoms (9 items) based on caregiver interview, the caregiver- and child-rated symptom checklists for attention-deficit/hyperactivity disorder (20 items) and disruptive behavior disorders (28 items), and the caregiver-rated symptom checklists for attachment disorders (ten items) and posttraumatic stress disorder (19 items). Emotion regulation strategies were examined using the Questionnaire for the Regulation of Frustration in children (FRUST); [35]. The FRUST captures caregiver-rated and child-rated adaptive emotion regulation strategies (10 and 33 items, respectively) and maladaptive emotion regulation strategies (4 and 7 items, respectively). We assessed quality of life using the child-rated KIDSCREEN-10-Index (10 items) and the caregiver-rated KIDSCREEN-27 (27 items); [36].

##### Caregiver characteristics

Caregiver AD symptoms were assessed using the Aggression and Hostility subscale (five items) from the German version of the Brief Symptom Checklist (BSCL); [37]. Moreover, a broad spectrum of psychopathology was examined using a short nine-item version of the German version of the Symptom Checklist (SCL-K-9;[38]). The Positive and Negative Parenting Questionnaire (FPNE; [39]) was administered to capture positive parenting behavior (13 items) and negative-inept parenting behavior (10 items). As potential protective factors, we measured family climate (nine items) using the Family Climate Scale (FCS); [40], social support (eight items) using an adapted short version of the Social Support Scale (SSS),[41], and personal resources (five items) using the Personal Resources Questionnaire (PRQ), [42].

##### COVID-19-related changes in external conditions

We analyzed the four items of the caregiver-rated total stress scale (CCSS-P-Stress) focusing on external conditions (childcare, working conditions, family conditions, leisure options) as potential risk factors for COVID-19-related stress.

In the current sample, all scales on risk and protective factors demonstrated sufficient to excellent internal consistencies (*α* = 0.73 to *α* = 0.97). For more details, see table S2.

### Data analysis

All statistical analyses were performed using SPSS 28 [43]. Missing data were imputed using the expectation maximization (EM) method on the item level for each scale separately if at least 90% of the items were available. Items of the respective scale were used for the imputation prediction.

Differences in sample characteristics between the participating and non-participating families and between the AD and No AD group were calculated using *χ*^2^ tests for categorical variables and *t*-tests for continuous variables. As a measure of effect size, we calculated Cohen`s d [44] for continuous variables and Phi for dichotomous variables. In line with Cohen [44], we used the following interpretations for *d*: 0.20 ≤ *d* ≤ 0.39 as small, 0.40 ≤ *d* ≤ 0.79 as moderate, *d* ≤ 0.80 as large, and the following interpretations for Phi: 0.10 ≤ *ϕ* ≤ 0.29 as small, 0.30 ≤ *ϕ* ≤ 0.49 as moderate, *ϕ* ≤ 0.50 as large.

In line with our first aim, we compared the caregiver- and child-rated COVID-19-related stress levels of the AD group with those of the No AD group, and the caregiver- and child-rated COVID-19-related stress levels of the subsamples with adoptive or biological caregivers with those of the subsamples from out-of-home care, based on child and caregiver report. The primary outcome was caregiver- and child-rated COVID-19-related stress on the scale level and the secondary outcome was caregiver- and child-rated COVID-19-related stress on the item level. Differences in COVID-19-related stress between two groups were calculated using Mann–Whitney *U* tests for the item level and *t*-tests for independent samples for the scale level. Differences in COVID-19-related stress between three groups were calculated using Kruskal–Wallis tests for the item level and one-way ANOVAs for independent samples for the scale level. One-way ANOVA contrasts were subsequently performed for significant differences between three subgroups. For pairwise comparisons, we used Cohen`s d as a measure of effect size using the interpretation mentioned above. For comparisons of three groups, we used partial eta squared, with the following interpretation [44]: 0.01 ≤ *ƞ*^2^_p_ ≤ 0.05 as small, 0.06 ≤ *ƞ*^2^_p_ ≤ 0.13 as moderate, *ƞ*^2^_p_ ≤ 0.14 as large.

In line with our second aim, we calculated the proportion of families describing improvements (− 2 or − 1 on the item level; − 2.00 to − 0.50 on the scale level) and deteriorations (0.50 or 2 on the item level; 0.05–2 on the scale level) on treatment-related items and subscales according to caregiver report.

In line with our third aim, the analysis of risk and protective factors of COVID-19-related stress, we used the child-rated total stress scale and the internal subscale of the caregiver-rated total stress scale as criterion in children with and without AD. Sociodemographic variables, child and caregiver characteristics, and perceived changes in external conditions were used as predictors. We developed the models for the AD group and the No AD group separately to be able to depict risk and protective factors in a more detailed way for each group. First, we calculated Pearson correlation coefficients for continuous variables and point-biserial correlation for dichotomous variables. Risk and protective factors were only included if the correlations with the respective criterion were significant (*p* < 0.05) and at least small (r ≥ 0.10); [44]. To avoid multicollinearity, the variable with the strongest correlation with the criterion was included in the case of very high correlations between predictors (*r* ≥ 0.80). To construct the final model, we used backward elimination, i.e., items were excluded stepwise from the initial model if they did not reach a significance level of at least *p* = 0.100. To evaluate the strength of predictors and the total model, we used Cohen`s *f*^2^ for the regression analyses with the following interpretation [44]: 0.02 ≤ *f*^22^ ≤ 0.14 as small, 0.15 ≤ *f*^2^ ≤ 0.34 as moderate, *f*^2^ ≤ 0.35 as large.

## Results

### Participant characteristics

Characteristics of participating and non-participating families and of the AD and No AD groups are displayed in Tables S3 and S4, respectively.

#### Participating and non-participating families

We did not find differences in retention between the AD group and the No AD group (*ϕ* = 0.05, ns) or between the out-of-home care subsamples and the subsamples with adoptive or biological caregivers (*ϕ* = 0.03, ns). Compared to non-participating families, participating families showed slightly lower levels of child attention problems (*d* = 0.26) and aggressive behavior (*d* = 0.20). The significant effects regarding child age, child affective dysregulation, caregiver education, and single parent status were negligible in size (*d* = 0.13–0.17; *ϕ* = 0.07). We did not find differences regarding child gender or child anxious/depressive symptoms.

#### AD and No AD groups

There was a slightly higher proportion of boys in the AD group compared to the No AD group (*ϕ* = 0.19). Additionally, caregivers reported slightly lower levels of education (*d* = 0.20) and considerably more symptoms of child AD (*d* = 3.35), anxiety/depression (*d* = 1.36), attention problems (*d* = 1.65), and aggression (*d* = 2.86). The significant effect regarding single parent status was negligible in size (ϕ = 0.08). We did not find differences regarding child age.

### Differences in COVID-19-related stress

Results of all comparisons on the scale level are shown in Table 1. Detailed analyses on the item level are provided in Table S5. We did not find any meaningful differences between the AD and the No AD group on the scale level, either in caregiver report (*d* = 0.18) or in child report (*d* = 0.09, ns). The COVID-19-related changes across domains were broadly comparable between the AD and the No AD group. However, small effects emerged for family relationships (*d* = 0.27) and family conditions (*d* = 0.28) in caregiver report, and for externalizing behavior (*d* = 0.28) in child report, with the AD group describing slightly higher stress levels than the No AD group.

**Table 1:** Differences between subsamples in COVID-19-related stress in caregiver and child report on the total stress scale

	Caregiver-rated stress				Child-rated stress			
	*n*	*M*	SD	Test statistics	*n*	*M*	SD	Test statistics
AD	263	0.48	0.66	*t(398)* = *2.16, p* = 0.031, *d* = 0.18	226	0.27	0.61	*t(341)* = 0.85, *p* = 0.394, *d* = 0.09
No AD	508	0.38	0.46		136	0.22	0.46	
AD								
Screened community_0-10_	169	0.48	0.65	*f*(2) = 0.10; *p* = 0.909, *ƞ*^2^_p_ < 0.01	145	0.23	0.64	*f*(2) = 1.36; *p* = 0.259, *ƞ*^2^_p_ = 0.01
Clinical	42	0.52	0.73		36	0.24	0.61	
Out-of-home care	52	0.47	0.64		45	0.40	0.48	
No AD								
Screened community_11-89_	361	0.40	0.47	*f*(2) = 1.63; *p* = 0.198, *ƞ*^2^_p_ = 0.01	0			
Screened community_0-10_	134	0.33	0.45		124	0.21	0.45	*t*(134) = 0.808; *p* = 0.421, *d* = 0.24
Out-of-home care	13	0.47	0.26		12	0.32	0.60	

*AD* sample with affective dysregulation, *No AD* sample without affective dysregulation, *Screened community*_*0-10*_ screened community sample with very low scores within the 10th percentile, *Screened community*_*11-89*_ screened community sample with typical or subclinical scores between the 11th and 89th percentile, *n* sample size, *M* mean, *SD* standard deviation, *t* *t*-test statistics, *f* one-way ANOVA statistics, *p* significance, *d* effect size Cohen’s d, *ƞ*^2^_p_ effect size partial eta squared

For children with AD, we did not find any meaningful differences on the scale level between the out-of-home care sample and the samples with adoptive or biological parents, either in caregiver report (*ƞ*^2^_p_ < 0.01, ns) or in child report (*ƞ*^2^_p_ = 0.01, ns). Again, the COVID-19-related changes across domains were broadly comparable. However, small effects emerged for working conditions in caregiver report (*ƞ*^2^_p_ = 0.02) and for child internalizing problems in child report (*ƞ*^2^_p_ = 0.05). Upon direct comparison, children in out-of-home care and their caregivers showed slightly higher levels of negative changes in working conditions compared to the community sample (*d* = 0.37) and considerably higher levels of internalizing problems compared to both the community sample (*d* = 0.71) and the clinical sample (*d* = 0.74). For children without AD, we did not find meaningful differences on the scale level between the out-of-home care sample and the samples with adoptive or biological parents, either in caregiver report (*ƞ*^2^_p_ = 0.01, ns) or in child report (*d* = 0.24, ns). Again, the COVID-19-related changes across domains were broadly comparable. However, small effects emerged for working conditions (*ƞ*^2^_p_ = 0.02). Upon direct comparison, caregivers of children in out-of-home care showed considerably higher levels of negative changes in working conditions compared to the screened community_11-89_ (*d* = 0.71). In addition, we found small effects between the two community samples for child externalizing problems (*ƞ*^2^_p_ = 0.02) and family conditions (*ƞ*^2^_p_ = 0.01) in caregiver report. Although small to moderate effect sizes emerged for differences between the community sample and the out-of-home care sample in child report (*d* = 0.31–0.54), these effects were not statistically significant.

### Treatment-related effects

On the scale level, only 18.3% of caregivers reported treatment-related deteriorations, while 19.5% reported improvements. For the domains use of therapeutic interventions and satisfaction with therapy, most caregivers reported no changes (62.6–90.5%). Improvements and deteriorations approximately balanced each other out. In terms of the individual problems of the child, just over a third reported no change, just over a third reported a slight deterioration, and just over a quarter reported improvements.

### Predictors of COVID-19-related stress

Table S8 presents the correlation analyses. There were no correlations above *r* = 0.80 for the relevant predictors. Therefore, we considered all relevant predictors for each model.

The final backward regression models for child-rated stress levels are presented in Table 2. We found moderate effect sizes for the final models in the AD group (*f*^2^ = 0.25) and the No AD group (*f*^2^ = 0.29). In the AD group, external conditions (family conditions, leisure options, childcare conditions), clinician-rated internalizing problems (assessed pre-pandemic), and school type were relevant predictors, explaining 20.2% of the variance. For the No AD group, the only relevant predictors were negative change in external conditions (family conditions, leisure options) and mother’s country of birth, explaining 17.8% of the variance. In both models, the effects of the aforementioned predictors were small.

**Table 2:** Reduced backward regression models for child-rated COVID-19-related stress in families of children with and without affective dysregulation

	AD (*n* = 183)				No AD (*n* = 121)			
	*ß*	*t*	*p*	*f*^2^	*ß*	*t*	*p*	*f*^2^
COVID-19-related negative change in								
Family conditions^a^	0.19	2.59	0.011	0.04	0.31	3.69	< 0.001	0.12
Leisure options^a^	0.18	2.49	0.014	0.04	0.20	2.43	0.017	0.05
Childcare conditions^a^	0.18	2.33	0.021	0.03				
Mother’s country of birth (1 = German, 2 = other than German)					0.14	1.67	0.097	0.02
Internalizing behavior^b^	0.17	2.47	0.015	0.03				
School type (1 = primary, 2 = secondary)	− 0.13	− 2.01	0.046	0.02				
corrected *R*^2^	20.2%				17.8%			
*f*^2^	0.29				0.25			

*AD* sample with affective dysregulation, *No AD* sample without affective dysregulation, *n* sample size, *ß* standardized coefficient, *t* *t*-test statistics, *p* significance, *f*^2^ effect size Cohen`s *f*^2^, *R*^2^ variance explained

^a^Caregiver-rated

^b^Clinician-rated

^c^Child-rated

The final backward regression models for child-rated stress levels are presented in Table 3. We found large effect sizes for the final models in the AD group (*f*^2^ = 1.32) and the No AD group (*f*^2^ = 0.40). In the AD group, external conditions (family conditions, leisure options, childcare conditions, working conditions), family adversity, and mother’s country of birth explained 55.7% of the variance. In the No AD group, the external conditions (family conditions, leisure options, childcare conditions), psychopathology of the caregiver, and child maladaptive emotion regulation strategies (both assessed pre-pandemic) explained 25.9% of the variance. Across all models, we mainly found small effects for the predictors. However, moderate effects emerged for family conditions (*f*^2^ = 0.20) and childcare conditions (*f*^2^ = 0.30) in the AD sample. Mother’s country of birth did not show a meaningful effect in the AD sample (*f*^2^ = 0.01).

**Table 3:** Reduced backward regression models for caregiver-rated COVID-19-related stress in families of children with and without affective dysregulation

	AD (*n* = 220)				No AD (*n* = 131)			
	*ß*	*t*	*p*	*f*^2^	*ß*	*t*	*p*	*f*^2^
COVID-19-related negative change in								
Family conditions^a^	0.34	6.51	< 0.001	0.20	0.20	2.53	0.013	0.05
Leisure options^a^	0.39	7.94	< 0.001	0.06	0.31	3.96	< 0.001	0.13
Childcare conditions^a^	0.18	3.43	0.001	0.30	0.17	2.16	0.033	0.04
Working conditions^a^	0.12	2.60	0.010	0.03				
Psychopathology of caregiver ^a^					0.19	2.42	0.017	0.05
Maladaptive emotion regulation ^b^					0.14	1.83	0.070	0.03
Family adversity ^c^	0.10	2.19	0.029	0.02				
Mother’s country of birth (1 = German, 2 = other than German)	0.08	1.77	0.077	0.01				
Corrected *R*^2^	55.7%				25.9%			
*f*^2^	1.32				0.40			

*AD* sample with affective dysregulation, *No AD* sample without affective dysregulation, *n* sample size, *ß* standardized coefficient, *t* *t*-test statistics, *p* significance, *f*^2^ effect size Cohen`s *f*^2^, *R*^2^ variance explained

^a^Caregiver-rated

^b^Child-rated

^c^Adversity based on the Family Adversity Index (cf. [30])

## Discussion

The present study investigated COVID-19-related stress in a sample of children with and without AD and their families. Only small differences emerged between families of children with and without AD or between children in out-of-home care and in adoptive/biological families: children with AD and children in out-of-home care were affected slightly more, but in few domains. Most families receiving therapy did not describe treatment-related changes. Perceived negative change in external conditions—particularly family conditions and leisure options—emerged as the most important risk factors for COVID-19-related stress across all models. When comparing families with and without AD and children in out-of-home care and in adoptive/biological families, no or only small differences emerged. Although some previous studies reported comparable findings (e.g. [10, 11, 45]), others indicated differences between families of children with and without mental health problems (e.g. [6, 46]). This might be explained by the timing of the respective surveys. Breaux et al. [6] found that the identified differences were only present during stay-at-home orders, suggesting that differences might be linked to the specific COVID-19-related restrictions, and disappeared after their removal. Moreover, families might have adapted to the new situation and developed new and necessary routines, particularly for children with special needs, which in turn may have led to a homogenization of the subgroups.

The latter assumption is supported by the fact that families scarcely reported any negative treatment-related effects, which was surprising given that psychotherapeutic care has been disrupted or limited for most families during the pandemic [19]. To limit any potential negative effects, therapies were reorganized into online sessions or a combination of online and face-to-face sessions as quickly as possible. In view of the common organizational barriers to face-to-face sessions (e.g.; logistical problems or childcare for siblings); [20], this reorganization might have alleviated strain, at least for some caregivers. Additionally, caregivers might have used the opportunity of more time with the family due to the COVID-19 measures [9] to practice and implement new therapeutic strategies in daily life more intensively.

As initially hypothesized, we found an association between COVID-19-related stress and measures of AD in the correlation analyses. However, in the regression models, these variables were excluded in favor of other predictors. The strongest risk factors across all models were COVID-19-related perceived changes in external conditions. Negative changes in family conditions and leisure options proved to be particularly stable predictors across all models. Prior to the outbreak of COVID-19, family routines were mostly set by work and school schedules, and potentially also by leisure activities. However, since all these areas were affected by restrictions, families may have struggled to restructure daily routines. As the present sample consisted of families living in and around larger cities, alternative leisure options might have been especially limited due to the urban living conditions (e.g.; noise regulations for families living in apartments, less space, no garden for physical activities). Moreover, the increased amount of time spent together might have increased the potential for family conflicts. On the other hand, some families may have perceived benefits from the fact that they did not have to adhere to structures set by others and were able to spend more quality time together. These families might have therefore perceived the circumstances as a positive change in family conditions, consequently leading to decreased stress levels.

Although we found small effects for the examined predictors in general, moderate effects emerged for the effect of family and childcare conditions on caregiver-rated stress in the AD group only, which may have led to the particularly large effect of the total model. Negative changes in both of these domains might place high demands on caregivers’ organizational skills and adaptability. As these demands may already be high in caregivers handling AD in daily life, the new external demands due to the pandemic may have exceeded the available resources more strongly in the caregivers in the AD group. Accordingly, external conditions should be considered when designing actions to support families to reduce stress, particularly regarding family conditions and leisure options, and additionally regarding childcare conditions for families with AD.

Caregiver characteristics (mother’s country of birth, psychopathology of caregiver, and family adversity) emerged as additional meaningful risk factors for both caregiver-reported stress (No AD and AD group) and child-reported stress (No AD group). These results correspond to studies demonstrating a buffering or aggravating effect of caregiver characteristics on COVID-19-related stress in children [22]. Interestingly, child characteristics (internalizing behavior and school type) only emerged as relevant risk factors for child-rated stress in the AD group, whereas in the No AD group, child characteristics (maladaptive emotion regulation) only emerged as risk factors for caregiver-rated stress. Children with AD typically lack effective use of emotion regulation strategies [15], and primary school children with AD may have struggled even more than secondary school children, as with age, emotion regulation usually increases and AD decreases [47]. Thus, these children might have had fewer resources to cope with the negative changes due to COVID-19, leading to increased stress levels [12]. The higher external demands due to COVID-19 may have made it especially difficult to cope with internalizing symptoms in addition to AD. Moreover, caregivers may not have recognized the child’s struggles, since internalizing symptoms are less visible than externalizing problems, and may, therefore, have been unable to provide adequate support. Conversely, caregivers of children without AD may have been accustomed to their child showing effective self-regulation, rendering maladaptive self-regulation with increased pandemic-related external demands more evident.

Some limitations of the present study should be mentioned. First, the interval for measuring COVID-19-related stress was rather long compared to other studies. While this enabled us to capture the prolonged stress reactions across different phases of COVID-19-related measures, it did not allow us to relate effects to specific pandemic-related measures (cf. [24]). Second, we compared ratings by parents of their adoptive or biological children with ratings by foster parents or professional caregivers in out-of-home care institutions. As most items of the total stress scale focused on the child, we consider this procedure as adequate. Nevertheless, there are some items that might be more significant for parents than for professional caregivers and vice versa. Third, we did not include objective measures of external conditions during COVID-19. A higher perceived negative change in external conditions could also have resulted from higher stress. Fourth, the No AD group in the out-of-home care sample was comparatively small (CCSS-P-Stress: *n* = 13; CCSS-C-Stress: *n* = 12). It is possible that the small to moderate differences between the out-of-home care sample and the community sample in child report might have reached the threshold for significance in a larger sample. To our knowledge, the present study is the first to include children in out-of-home care, and the results should, therefore, be interpreted as first indications of the development of COVID-related stress in this sample. Fifth, despite a comparatively high response rate of 60% overall, we found small differences between participating and non-participating families. In particular, parents in non-participating families reported more externalizing behavior in their children. Therefore, we cannot rule out some bias. Potentially, COVID-19-related effects on stress levels might have been more extreme in the total sample. Finally, even though we were able to analyze a variety of potential predictors, there may be other relevant risk or protective factors that were not considered within our study, e.g.; couple conflict or fear/worry about COVID-19 [24].

Nevertheless, several strengths of the study are also worthy of mention. First, we recruited a diverse sample of children with and without AD and a combination of screened community, clinical, and out-of-home care samples. Second, we included caregiver- and child-reported measures to assess COVID-19-related factors. Third, to assess caregiver and child characteristics as potential risk and protective factors, we used previously captured data, suggesting a causal link within the regression analysis. Finally, we included a variety of established reliable and valid child-rated, caregiver-rated, and clinician-rated instruments.

Taken together, our findings indicate that the changes rated by children and caregivers were overall comparable between families of children with and without AD and between children in out-of-home care and in adoptive or biological families. The strongest and most stable predictors of increased stress overall were perceived negative changes in external conditions—particularly family conditions and leisure options. Actions to support families during the pandemic should, therefore, focus on these factors to reduce COVID-19-related stress.

## Supplementary Information

Below is the link to the electronic supplementary material.

Supplementary file1 (DOCX 74 KB)

## Data Availability

The dataset can be obtained from the corresponding author after publication of the main outcome analyses on treatment effects.
