# Supplementary material for: Impact of the COVID-19 pandemic on children with and without affective dysregulation and their families
Source: Eur Child Adolesc Psychiatry. 2022 Nov 16;32(6):951–61. doi: 10.1007/s00787-022-02106-3 (PMC9668221; doi:10.1007/s00787-022-02106-3)
Supplement: Supplementary file 1 — Supplementary file1 (DOCX 74 KB) [file 787_2022_2106_MOESM1_ESM.docx]

**Supplementary material**

**Impact of the COVID-19 pandemic on children with and without affective dysregulation and their families**

Treier, A.-K.^1,2^, Holas, V.^1,2^, Görtz-Dorten, A.^1,2^, Frenk, F. ^1,2^, Goldbeck, C. ^1,2^, Mücke, K. ^1,2^, Hanisch, C.^3^, Ritschel, A.^3^, Roessner, V.^4^, Rothe, J. ^4^, Ravens-Sieberer, U.^5^, Kaman, A.^5^, Banaschewski, T.^6^, Brandeis, D.^6,7,8,9^, Aggensteiner, P.-M. ^6^, Kölch, M.^10,11^, Daunke, A.^10^, Döpfner, M.^1,2^ for the ADOPT Consortium

^1^Department of Child and Adolescent Psychiatry, Psychosomatics and Psychotherapy, Medical Faculty of the University of Cologne, Cologne, Germany

^2^School for Child and Adolescent Cognitive Behavior Therapy (AKiP), Medical Faculty of the University of Cologne, Cologne, Germany

^3^Faculty of Human Sciences, University of Cologne, Cologne, Germany

^4^Department of Child and Adolescent Psychiatry and Psychotherapy, TU Dresden, Dresden, Germany

^5^Department of Child and Adolescent Psychiatry, Psychotherapy, and Psychosomatics & Research Unit Child Public Health, University Medical Center Hamburg-Eppendorf, Hamburg, Germany

^6^Department of Child and Adolescent Psychiatry and Psychotherapy, Central Institute of Mental Health, Medical Faculty Mannheim, University of Heidelberg, Mannheim, Germany

^7^Department of Child and Adolescent Psychiatry and Psychotherapy, Psychiatric Hospital, University of Zürich, Zürich, Switzerland.

^8^Zürich Center for Integrative Human Physiology, University of Zürich, Zürich, Switzerland.

^9^Neuroscience Center Zürich, University and ETH Zürich, Zürich, Switzerland.

^10^Department of Child and Adolescent Psychiatry, Neurology, Psychosomatics, and Psychotherapy, University Medical Center Rostock, Rostock, Germany

^11^Department of Child and Adolescent Psychiatry/Psychotherapy, University of Ulm, Ulm, Germany

Correspondence: anne-katrin.treier@uk-koeln.de**Figure S1**

*Flowchart depicting the COVID-19 study sample*

**Note:** AD = sample with affective dysregulation. No AD = sample without affective dysregulation. **Table S1**

*Items of the caregiver-rated version of the Corona Child Stress Scale in caregiver (CCSS-P) and child rating (CCSS-C)*

| **No.** | **short term** | **item in caregiver rating** | **item in child rating** |
| --- | --- | --- | --- |
|  |  | **caregiver-rated total stress scale (CCSS-P-Stress)** | **child-rated total stress scale (CCSS-C-Stress)** |
| 1 | family relationships | Have the relationships between your child and the other family members changed due to COVID-19? | Have the relationships between you and the other family members changed due to COVID-19 (e.g., how you and your mother treat each other, how you get along with your father, how you get along with your siblings)? |
| 2 | peer relationships | Have the relationships between your child and his/her friends changed due to COVID-19? | Have the relationships between you and your friends changed due to COVID-19 (e.g., how much contact you have with your friends or how you get along)? |
| 3 | school stress | Has your child’s burden regarding school and learning changed due to COVID-19? | Has your burden regarding school and learning changed due to COVID-19 (e.g., how you do schoolwork and how much you learn)? |
| 4 | childcare conditions | Has your childcare (apart from school) changed due to COVID-19? | x |
| 5 | leisure options | Have the leisure options for your child changed due to COVID-19? | Have your leisure options changed due to COVID-19 (e.g., how much you can do, whether you can pursue your hobbies or interests, whether you can meet up with others)? |
| 6 | child externalizing problems | Is your child more irritable, moody, or restless due to the COVID-19 crisis? | Are you more irritable, moody, or restless due to the COVID-19 crisis? |
| 7 | child internalizing problems | Is your child more anxious, insecure, or sad due to the COVID-19 crisis? | Are you more anxious, insecure, or sad due to the COVID-19 crisis? |
| 8 | working conditions | Has your work situation changed due to the COVID-19 crisis? | x |
| 9 | family conditions | Has your family situation changed due to the COVID-19 crisis? | x |
| 10 | child stress overall | How would you describe the changes due to the COVID-19 crisis for your child in general? | How have you felt in the COVID-19 crisis in general? |
| 11 | caregiver stress overall | How would you describe the changes due to the COVID-19 crisis for yourself in general? | x |
|  |  | **caregiver-rated treatment-related subscale (CCSS-P-Treat)** | **child-rated treatment-related subscale (CCSS-C-Treat)** |
| 12 | change in individual problems | How have the individual problems of your child, which were the main motive for therapy, changed during the COVID-19 crisis? | x |
| 13 | use of therapeutic interventions | Has the extent to which you or your child implements strategies taught in therapy changed due to COVID-19 crisis? | Has the extent to which you implement strategies taught in therapy changed due to COVID-19 crisis (e.g., how you have used or practiced the contents of therapy in daily life)? |
| 14 | satisfaction with therapy | Has your satisfaction with therapy changed due to the COVID-19 crisis? | Has your satisfaction with therapy changed due to the COVID-19 crisis? |

**Table S2**

*Measures assessing child and caregiver characteristics used as predictors of COVID-19-related stress*

| **variable(s)** | **measure** | **type** | **content** | **scale** | **alpha** |
| --- | --- | --- | --- | --- | --- |
| Child affective dysregulation (AD) | *Diagnostic Tool for Affective Dysregulation in Children* (DADYS; Anja Görtz-Dorten & Manfred Döpfner, 2021) | Clinical interview for caregivers and children, caregiver- and child-rated questionnaire | Emotional lability, emotion regulation, negative emotional reactions, negative mood | 4-point scale: 0 (=not present) to 3 (=very strong) | .86-.97 |
| Child AD | Dysregulation profile (Althoff, 2010) of the German version of the *Child Behavior Checklist* (CBCL/6-18R; Döpfner et al., 2014) | Caregiver-rated questionnaire | *Anxious/depressive symptoms, attention problems, aggressive behavior* | 3-point scale: 0 (= not true) to 2 (= very true or often true) | .82-.94 |
| Child internalizing, externalizing symptoms | Diagnostic System for Mental Disorders in children and adolescents according to ICD-10 and DSM-5, DISYPS-III (ICD-10 and DSM-IV; Döpfner & Görtz-Dorten, 2016). | Clinical interview for caregivers, caregiver- and child-rated questionnaire | Internalizing and externalizing symptoms, attention-deficit/ hyperactivity disorder, disruptive behavior, attachment disorders, posttraumatic stress disorder | 4-point scale: 0 (=age-typical) to 3 (=very strong) | .75-.96 |
| Emotion regulation of child | Questionnaire for the Regulation of Frustration (FRUST; A. Görtz-Dorten & M. Döpfner, 2021) | Caregiver- and child-rated questionnaire | Adaptive and maladaptive emotion regulation strategies | 5-point scale: 0 (=hardly ever) to 4 (almost always) | .73-.94 |
| Quality of life of child | KIDSCREEN-10-Index, KIDSCREEN-27 (The KIDSCREEN Group Europe, 2006) | Caregiver- and child-rated questionnaire | Subjective health and well-being | 5-point scale: 1 (=never/not at all) to 5 (=always/very strong) | .78-.91 |
| AD in caregiver | German version of the Brief Symptom Checklist (BSCL; Franke, 2017) | Caregiver-rated questionnaire | Aggression and hostility | 5-point scale: 0 (=not at all) to 4 (=very strong) | .75 |
| Psychopathology of caregiver | German version of the Symptom Checklist (SCL-K-9; Klaghofer & Brähler, 2001) | Caregiver-rated questionnaire | Global psychological distress | 5-point scale: 1 (=not at all) to 5 (=very strong) | .84 |
| Parenting behavior | Positive and Negative Parenting Questionnaire (FPNE; Holas et al., 2021) | Caregiver-rated questionnaire | Positive and negative-inept parenting behavior | 4-point scale: 1 (=never) to 4 (=very often) | .81-.87 |
| Protective family factors | Family Climate Scale (FCS; Schneewind et al., 1985) | Caregiver-rated questionnaire | Family climate | 4-point scale: 1 (=not right at all) to 4 (=exactly right) | .76 |
|  | short version of the Social Support Scale (SSS; Sherbourne & Stewart, 1991) | Caregiver-rated questionnaire | Social support | 5-point scale: 1 (=never) to 5 (=always) | .89 |
|  | Personal Resources Questionnaire (PRQ; cf. Holling & Schlack, 2008) | Caregiver-rated questionnaire | Personal resources | 4-point scale: 1 (=not right at all) to 4 (=exactly right) | .87 |

**Table S3**

*Participant and non-participant characteristics of the COVID-19 assessment*

|  | non-participating families  (*n*=527) | participating families  (*n*=781) | test statistic | *p* | effect size |
| --- | --- | --- | --- | --- | --- |
| child variables |  |  |  |  |  |
| age (years): *M* (*SD*) | 10.36 (1.49) | 10.16 (1.47) | *t*(1295)=2.30 | .011 | *d*=0.13 |
| gender (male): % | 56.2 | 54.7 | χ²(1)=0.28 | .594 | ϕ=0.01 |
| affective dysregulation: *M* (*SD*)^a^ | 0.80 (0.61) | 0.71 (0.58) | *t*(1024)=-2.65 | .004 | *d*=0.15 |
| anxious/depressed^b^ | 0.26 (0.28) | 0.25 (0.27) | *t*(1065)=-1.05 | .146 | *d*=0.06 |
| attention problems^b^ | 0.46 (0.46) | 0.35 (0.40) | *t*(1005)=-4.48 | <.001 | *d*=0.26 |
| aggressive behavior^b^ | 0.45 (0.47) | 0.36 (0.41) | *t*(998)=-3.39 | .001 | *d*=0.20 |
| caregiver variables |  |  |  |  |  |
| education^c^: *M* (*SD*) | 6.00 (1.38) | 6.22 (1.25) | *t*(900)=2.82 | .004 | *d*=0.17 |
| single parent status: % | 14..9 | 12.2 | χ²(1)=5.40 | .020 | ϕ=0.07 |

**Note:** *n* = sample size. *p* = significance. *M*=mean. *SD*=standard deviation. *t* = t-test statistics. χ² = χ² test statistics. *d* = effect size Cohen’s d. ϕ = effect size Phi.

^a^caregiver-rated questionnaire of the Diagnostic Tool for Affective Dysregulation in Children

^b^caregiver-rated Child Behavior Checklist

^c^highest educational level within family based on Lampert, Hoebel, Kuntz, Müters, Kroll (2018)

**Table S4**

*Participant characteristics at pretreatment for children with and without affective dysregulation*

|  | No AD  (*n*=512) | AD  (*n*=269) | test statistic | *p* | effect size |
| --- | --- | --- | --- | --- | --- |
| child variables |  |  |  |  |  |
| age^a^ (years): *M* (*SD*) | 11.61 (1.44) | 11.51 (1.56) | *t*(769)=.876 | .191 | *d*=0.07 |
| gender (male): % | 47.9 | 67.7 | χ²(1)=27.92 | <.001 | ϕ=0.19 |
| affective dysregulation ^b^: *M* (*SD*) | 0.36 (0.24) | 1.39 (0.40) | *t*(371)=-38.36 | <.001 | *d*=3.35 |
| anxious/depressed^c^ | 0.14 (0.15) | 0.45 (0.33) | *t*(326)=-14.70 | <.001 | *d*=1.36 |
| attention problems^c^ | 0.18 (0.23) | 0.69 (0.42) | *t*(356)=-18.56 | <.001 | *d*=1.65 |
| aggressive behavior^c^ | 0.12 (0.15) | 0.81 (0.35) | *t*(324)=-30.72 | <.001 | *d*=2.86 |
| caregiver variables |  |  |  |  |  |
| education^d^: *M* (*SD*) | 6.29 (1.18) | 6.04 (1.40) | *t*(378)=2.32 | .021 | *d*=0.20 |
| single parent status: % | 8.8 | 14.1 | χ²(1)=4.68 | .031 | ϕ=0.08 |

**Note:** AD = sample with affective dysregulation. No AD = sample without affective dysregulation. *n* = sample size. *p* = significance. *M*=mean. *SD*=standard deviation. *t* = t-test statistics. χ² = χ² test statistics. *d* = effect size Cohen’s d. ϕ = effect size Phi.

^a^at COVID-19 assessment

^b^caregiver-rated questionnaire of the Diagnostic Tool for Affective Dysregulation in Children

^c^caregiver-rated subscales of the Child Behavior Checklist (CBCL/6-18R)

^d^highest educational level within family based on Lampert, Hoebel, Kuntz, Müters, Kroll (2018)

**Table S5**

*Caregiver- and child-rated COVID-19-related stress* *in families of children with and without affective dysregulation on the item level*

|  | **caregiver-rated stress** | | | | | | **child-rated stress** | | | | |
| --- | --- | --- | --- | --- | --- | --- | --- | --- | --- | --- | --- |
|  | AD | | No AD | | test statistics | AD | | | No AD | | test statistics |
|  | *M* | *SD* | *M* | *SD* |  | *M* | | *SD* | *M* | *SD* |  |
| 1 family relationships | -0.02 | 1.01 | -0.23 | 0.66 | *U*=57791.00, *p*<.001, *d*=0.27 | 0.01 | | 0.90 | -0.10 | 0.72 | *U*=13997.50, *p*=.123, *d*=0.14 |
| 2 peer relationships | 0.47 | 0.79 | 0.37 | 0.68 | *U*=63161.50, *p*=.141, *d*=0.13 | 0.29 | | 0.93 | 0.25 | 0.87 | *U*=15138.00; *p*=.797, *d*=0.05 |
| 3 school stress | 0.40 | 1.19 | 0.27 | 0.99 | *U*=62172.50, *p*=.083, *d*=0.12 | 0.21 | | 1.08 | 0.15 | 0.92 | *U*=14659.00; *p*=.441, *d*=0.06 |
| 4 childcare conditions | 0.55 | 1.02 | 0.51 | 0.88 | *U*=65095.00, *p*=.509, *d*=0.04 |  | |  |  |  |  |
| 5 leisure options | 1.10 | 1.08 | 1.24 | 0.80 | *U*= 65858.50, *p*=.656, *d*=0.15 | 0.72 | | 1.18 | 0.87 | 0.87 | *U*=14886.50; *p*=.598, *d*=0.14 |
| 6 child externalizing problems | 0.44 | 1.15 | 0.28 | 0.87 | *U*=59089.50, *p*=.005, *d*=0.16 | 0.29 | | 0.95 | 0.10 | 0.75 | *U*=13123.00; *p*=.012, *d*=0.21 |
| 7 child internalizing problems | 0.19 | 0.72 | 0.18 | 0.60 | *U*= 65729.00, *p*=.621, *d*<0.01 | 0.09 | | 0.75 | 0.10 | 0.57 | *U*=14959.00; *p*=.597, *d*=0.02 |
| 8 working conditions | 0.45 | 0.91 | 0.32 | 0.82 | *U*=61828.50, *p*=.053, *d*=0.15 |  | |  |  |  |  |
| 9 family conditions | 0.18 | 0.98 | -0.05 | 0.72 | *U*= 56840.50, *p*<.001, *d*=0.28 |  | |  |  |  |  |
| 10 child stress overall | 0.65 | 1.14 | 0.53 | 0.95 | *U*= 60224.50, *p*=.016, *d*=0.12 | 0.25 | | 1.02 | 0.15 | 0.91 | *U*=14456.00; *p*=.320, *d*=0.10 |
| 11 caregiver stress overall | 0.89 | 1.09 | 0.76 | 1.04 | *U*= 61007.00, *p*=.034, *d*=0.12 |  | |  |  |  |  |
| 12 change in individual problems | 0.11 | 0.98 |  |  |  |  | |  |  |  |  |
| 13 use of therapeutic interventions | 0.09 | 0.74 |  |  |  | -0.01 | | 0.59 |  |  |  |
| 14 satisfaction with therapy | <0.01 | 0.61 |  |  |  | 0.06 | | 0.43 |  |  |  |

**Note:** AD = sample with affective dysregulation. No AD = sample without affective dysregulation. *M*=mean. *SD*=standard deviation. *U* = Mann-Whitney U test statistics. *p* = significance. *d* = effect size Cohen’s d. Caregiver-rated stress: *n_AD_*=263 for items 1 to 11. *n_AD_*=236 for item 12. *n_AD_*=182 for items 13 to 14. *n_NoAD_*=510. Child-rated stress: *n_AD_*=226 for items 1 to 10. *n_AD_*=158 for items 13 to 14. *n_NoAD_*=136. Test statistics are based on Mann-Whitney U tests for domain levels and t-tests for unpaired samples for scale levels.

**Table S6**

*Caregiver- and child-rated COVID-19-related stress in subsamples of families of children with affective dysregulation on the item level*

|  | **caregiver-rated stress** | | | | | | | **child-rated stress** | | | | | | |
| --- | --- | --- | --- | --- | --- | --- | --- | --- | --- | --- | --- | --- | --- | --- |
|  | screened community_0-10_ | | clinical | | out-of-home care | | test statistics | screened community_0-10_ | | clinical | | out-of-home care | | test statistics |
|  | *M* | *SD* | *M* | *SD* | *M* | *SD* |  | *M* | *SD* | *M* | *SD* | *M* | *SD* |  |
| 1 family relationships | -0.05 | 1.00 | 0.02 | 1.07 | 0.04 | 1.01 | χ²(2)=0.12; *p*=.944, ƞ²_p_<0.01 | -0.03 | 0.94 | <0.01 | 0.99 | 0.16 | 0.71 | χ²(2)=0.89; *p*=.639, ƞ²_p_=0.01 |
| 2 peer relationships | 0.49 | 0.80 | 0.48 | 0.83 | 0.40 | 0.75 | χ²(2)=1.22; *p*=.545, ƞ²_p_<0.01 | 0.21 | 0.94 | 0.31 | 1.06 | 0.56 | 0.76 | χ²(2)=3.79; *p*=.151, ƞ²_p_=0.02 |
| 3 school stress | 0.38 | 1.20 | 0.43 | 1.19 | 0.44 | 1.19 | χ²(2)=0.13; *p*=.939, ƞ²_p_<0.01 | 0.28 | 1.10 | 0.31 | 0.95 | -0.07 | 1.07 | χ²(2)=3.93; *p*=.140, ƞ²_p_=0.02 |
| 4 childcare conditions | 0.59 | 1.06 | 0.55 | 1.02 | 0.44 | 0.92 | χ²(2)=1.17; *p*=.557, ƞ²_p_<0.01 |  |  |  |  |  |  |  |
| 5 leisure options | 1.21 | 0.99 | 1.05 | 1.19 | 0.77 | 1.21 | χ²(2)=5.76; *p*=.056, ƞ²_p_=0.03 | 0.71 | 1.20 | 0.56 | 1.34 | 0.89 | 0.98 | χ²(2)=0.81; *p*=.666, ƞ²_p_=0.01 |
| 6 child externalizing problems | 0.44 | 1.15 | 0.50 | 1.17 | 0.38 | 1.16 | χ²(2)=0.40; *p*=.820, ƞ²_p_<0.01 | 0.19 | 0.99 | 0.47 | 0.94 | 0.47 | 0.82 | χ²(2)=4.50; *p*=.106, ƞ²_p_=0.02 |
| 7 child internalizing problems | 0.20 | 0.66 | 0.10 | 0.79 | 0.23 | 0.83 | χ²(2)=0.19; *p*=.911, ƞ²_p_<0.01 | 0.03 | 0.72 | -0.08 | 0.81 | 0.42 | 0.69 | χ²(2)=11.46; *p*=.003, ƞ²_p_=0.05 |
| 8 working conditions | 0.40 | 0.89 | 0.33 | 1.14 | 0.71 | 0.75 | χ²(2)=6.71; *p*=.035, ƞ²_p_=0.02 |  |  |  |  |  |  |  |
| 9 family conditions | 0.11 | 0.93 | 0.17 | 0.96 | 0.42 | 1.11 | χ²(2)=4.15; *p*=.125, ƞ²_p_=0.02 |  |  |  |  |  |  |  |
| 10 child stress overall | 0.62 | 1.13 | 1.00 | 1.01 | 0.48 | 1.21 | χ²(2)=5.85; *p*=.054, ƞ²_p_=0.02 | 0.23 | 1.02 | 0.14 | 1.15 | 0.38 | 0.91 | χ²(2)=0.71; *p*=.700, ƞ²_p_=0.01 |
| 11 caregiver stress overall | 0.85 | 1.15 | 1.12 | 1.09 | 0.83 | 0.81 | χ²(2)=4.79; *p*=.091, ƞ²_p_=0.01 |  |  |  |  |  |  |  |
| 12 change in individual problems | 0.12 | 1.01 | <0.01 | 1.26 | 0.14 | 0.81 | χ²(2)=0.24; *p*=.887, ƞ²_p_<0.01 |  |  |  |  |  |  |  |
| 13 use of therapeutic interventions | 0.13 | 0.74 | <0.01 | 0.98 | -0.04 | 0.47 | χ²(2)=1.42; *p*=.491, ƞ²_p_<0.01 | -0.03 | 0.59 | 0.25 | 0.55 | -0.15 | 0.59 | χ²(2)=5.74; *p*=.057, ƞ²_p_=0.03 |
| 14 satisfaction with therapy | <0.01 | 0.63 | 0.14 | 0.71 | -0.13 | 0.34 | χ²(2)=2.15; *p*=.342, ƞ²_p_=0.01 | 0.03 | 0.36 | 0.25 | 0.72 | 0.10 | 0.45 | χ²(2)=3.84; *p*=.147, ƞ²_p_=0.03 |

**Note:** *M*=mean. *SD*=standard deviation. χ² = Kruskal-Wallis test statistics. *p* = significance. ƞ²_p_ = effect size partial eta squared. Caregiver-rated stress: *n_community II_*=169 for items 1 to 11. *n_community 0-10_*=170 for item 12. *n_community 0-10_*=137 for items 13 to 14. *n_clinical_*=42 for items 1 to 11. *n_clinical_*=16 for item 12. *n_clinical_*=22 for items 13 to 14. *n_out-of-home care_*=52 for items 1 to 11. *n_out-of-home care_*=50 for item 12. *n_out-of-home care_*=23 for items 13 to 14. Child-rated stress: *n_community 0-10_*=145 for items 1 to 10. *n_community 0-10_*=118 for items 13 to 14. *n_clinical_*=36 for items 1 to 10. *n_clinical_*=20 for items 13 to 14. *n_out-of-home care_*=45 for items 1 to 10. *n_out-of-home care_*=20 for items 13 to 14. Test statistics are based on Kruskal-Wallis tests for domain levels and one-way ANOVAs for scale levels. **Table S7**

*Caregiver- and child-rated COVID-19-related stress in subsamples of families of children without affective dysregulation on the item level*

|  | **caregiver-rated stress** | | | | | | | | **child-rated stress** | | | | | |
| --- | --- | --- | --- | --- | --- | --- | --- | --- | --- | --- | --- | --- | --- | --- |
|  | screened community_11-89_ | | screened community_0-10_ | | out-of-home care | | | test statistics | screened community_0-10_ | | out-of-home care | | | test statistics |
|  | *M* | *SD* | *M* | *SD* | *M* | *SD* |  | | *M* | *SD* | *M* | *SD* |  | |
| 1 family relationships | -0.22 | 0.67 | -0.29 | 0.64 | 0.08 | 0.49 | χ²(2)=4.58; *p*=.101, ƞ²_p_=0.01 | | -0.14 | 0.73 | 0.25 | 0.62 | *U*=532.50; *p*=.064, d=0.54 | |
| 2 peer relationships | 0.34 | 0.69 | 0.44 | 0.64 | 0.69 | 0.63 | χ²(2)=4.75; *p*=.093, ƞ²_p_=0.01 | | 0.28 | 0.80 | -0.08 | 1.38 | *U*=617.50; *p*=.293, d=0.42 | |
| 3 school stress | 0.30 | 0.96 | 0.18 | 1.06 | 0.54 | 0.88 | χ²(2)=2.08; *p*=.354, ƞ²_p_=0.01 | | 0.11 | 0.91 | 0.58 | 0.90 | *U*=526.50; *p*=.078, d=0.52 | |
| 4 childcare conditions | 0.54 | 0.90 | 0.46 | 0.86 | 0.31 | 0.48 | χ²(2)=2.60; *p*=.272, ƞ²_p_<0.01 | |  |  |  |  |  | |
| 5 leisure options | 1.25 | 0.80 | 1.23 | 0.78 | 1.08 | 0.64 | χ²(2)=1.49; *p*=.474, ƞ²_p_<0.01 | | 0.88 | 0.82 | 0.75 | 1.29 | *U*=738.00; *p*=.959, d=0.15 | |
| 6 child externalizing problems | 0.36 | 0.88 | 0.07 | 0.81 | 0.31 | 1.03 | χ²(2)=12.95; *p*=.002, ƞ²_p_=0.02 | | 0.08 | 0.72 | 0.33 | 1.07 | *U*=586.00; *p*=.166, d=0.34 | |
| 7 child internalizing problems | 0.20 | 0.62 | 0.14 | 0.58 | 0.15 | 0.38 | χ²(2)=1.20; *p*=.548, ƞ²_p_<0.01 | | 0.10 | 0.52 | 0.17 | 1.03 | *U*=678.00; *p*=.504, d=0.12 | |
| 8 working conditions | 0.27 | 0.81 | 0.41 | 0.81 | 0.85 | 0.80 | χ²(2)=7.63; *p*=.022, ƞ²_p_=0.02 | |  |  |  |  |  | |
| 9 family conditions | -0.03 | 0.71 | -0.09 | 0.69 | <0.01 | 1.15 | χ²(2)=1.93; *p*=.381, ƞ²_p_<0.01 | |  |  |  |  |  | |
| 10 child stress overall | 0.57 | 0.96 | 0.43 | 0.94 | 0.54 | 0.66 | χ²(2)=2.67; *p*=.263, ƞ²_p_=0.01 | | 0.15 | 0.93 | 0.25 | 0.75 | *U*=700.50; *p*=.724, d=0.12 | |
| 11 caregiver stress overall | 0.82 | 1.04 | 0.60 | 1.06 | 0.62 | 0.51 | χ²(2)=7.26; *p*=.027, ƞ²_p_=0.01 | |  |  |  |  |  | |

**Note:** *M*=mean. *SD*=standard deviation. χ² = Kruskal-Wallis test statistics. *U* = Mann-Whitney U test statistics. *p* = significance. ƞ²_p_ = effect size partial eta squared. *d* = effect size Cohen’s d. Caregiver-rated stress: *n_community 11-89_*=363. *n_community 0-10_*=134. *n_out-of-home care_*=13. Child-rated stress: *n_community 0-10_*=124. *n_out-of-home care_*=12. Test statistics for three groups are based on Kruskal-Wallis tests for domain levels and one-way ANOVAs for scale levels. Test statistics for two groups are based on Mann-Whitney U tests for domain levels and t-tests for unpaired samples for scale levels.

**Table S8**

*Correlation analyses of potential predictors of caregiver- and child-rated COVID-19-related stress in families of children with and without affective dysregulation*

|  |  |  | **child-rated stress** | | **caregiver-rated internal stress** | |
| --- | --- | --- | --- | --- | --- | --- |
|  |  |  | **No AD** | **AD** | **No AD** | **AD** |
|  |  | **rater** | *n*=80-136 | *n*=134-226 | *n*=133-508 | *n*=152-263 |
| Sociodemographic | gender child |  | -.01 | .06 | -.01 | .04 |
|  | age child |  | -.10 | -.07 | -.06 | -.07 |
|  | age mother |  | -.07 | .05 | .01 | -.07 |
|  | age father |  | .03 | -.03 | .04 | -.07 |
|  | country of birth child^1^ |  | <.01 | .01 | -.03 | .01 |
|  | country of birth mother^1^ |  | .28^**^ | .13 | .10^*^ | .14* |
|  | country of birth father^1^ |  | .06 | .12 | .05 | .05 |
|  | native language^1^ |  | .15 | .01 | .07 | .06 |
|  | migration background^2^ |  | .17 | .04 | .07 | .07 |
|  | school type^3^ |  | -.04 | -.17^*^ | -.05 | -.11 |
|  | school grade |  | -.10 | -.08 | -.08 | -.04 |
|  | special educational needs^4^ |  | .04 | .15^*^ | <.01 | .11 |
|  | number of biological parents |  | -.11 | -.09 | -.08 | .01 |
|  | single parent status^4^ |  | .14 | .02 | -.04 | .06 |
|  | education mother^5^ |  | -.03 | .07 | <.01 | .01 |
|  | education father^5^ |  | .09 | .01 | -.03 | -.07 |
|  | occupation mother^5^ |  | -.01 | .03 | .08 | -.07 |
|  | occupation father^5^ |  | .02 | .02 | -.01 | -.02 |
|  | family adversity^6^ |  | .11 | .03 | .05 | .17* |

| child characteristics | AD | Clin (P) | .02 | .19^**^ | .05 | .04 |
| --- | --- | --- | --- | --- | --- | --- |
|  | AD | Clin (C) | .10 | .11 | .05 | -.08 |
|  | AD | P | .15 | .18^**^ | .11^*^ | .09 |
|  | AD | C | .13 | .07 | .20^*^ | -.03 |
|  | AD: anxious/depressed | P | .05 | .11 | .11^*^ | .10 |
|  | AD: attention problems | P | .06 | .09 | .07 | .04 |
|  | AD: aggressive behavior | P | .13 | .10 | .13** | .05 |
|  | externalizing behavior | Clin (P) | .01 | .10 | .08 | .09 |
|  | internalizing behavior | Clin (P) | .04 | .18^**^ | .05 | .10 |
|  | ADHD | P | .03 | .08 | .08 | .07 |
|  | ADHD | C | .07 | .11 | .12 | .01 |
|  | ODD | P | .14 | .08 | .08 | .07 |
|  | ODD | C | .09 | <.01 | .13 | -.03 |
|  | RAD | P | .15 | .09 | .05 | <.01 |
|  | DSED | P | .10 | -.09 | .08 | -.08 |
|  | PTSD | P | .11 | .08 | .06 | .08 |
|  | adaptive emotion regulation | P | .06 | -.01 | <.01 | -.01 |
|  | adaptive emotion regulation | C | -.16 | -.10 | -.19^*^ | .03 |
|  | maladaptive emotion regulation | P | -.02 | .11 | -.01 | .02 |
|  | maladaptive emotion regulation | C | .06 | <.01 | .17* | -.05 |
|  | quality of life | P | -.06 | -.11 | -.06 | -.04 |
|  | quality of life | C | -.18^*^ | -.11 | -.19* | <.01 |

| caregiver characteristics | AD in caregiver | P | .18^*^ | .04 | .14^**^ | .13 |
| --- | --- | --- | --- | --- | --- | --- |
|  | psychopathology of caregiver | P | .15 | .04 | .20** | .19** |
|  | positive parenting | P | -.10 | -.02 | -.08 | -.02 |
|  | negative-inept parenting | P | .03 | -.01 | .05 | .04 |
|  | family climate | P | -.11 | <.01 | -.11* | -.02 |
|  | social support | P | -.08 | -.07 | -.08 | -.06 |
|  | personal resources | P | .07 | -.13 | -.05 | -.05 |
| External (COVID-19) | childcare conditions | P | .11 | .29** | .31** | .40** |
|  | leisure options | P | .23** | .25** | .33** | .52** |
|  | working conditions | P | .10 | .19** | .29** | .31** |
|  | family conditions | P | .31** | .34** | .36** | .54** |

**Note:** AD = sample with affective dysregulation. No AD = sample without affective dysregulation. N = sample size. AD=affective dysregulation. ADHD=attention-deficit/hyperactivity disorder. ODD=oppositional defiant disorder. RAD=reactive attachment disorder. DSED=disinhibited social engagement disorder PTSD=posttraumatic stress disorder. C=child rating. P= caregiver rating. Clin (P)=clinical rating based on caregiver interview. Clin (C)=clinical rating based on child interview.

***p*< 0.01

**p*<0.05

^1^ 1=German, 2=other than German

^2^ native, first generation, second generation, as in PISA (OECD, 2017)

^3^ 1=primary school, 2=secondary school

^4^ 0=yes, 1=no

^5^ based on Lampert, Hoebel, Kuntz, Müters, Kroll (2018)

^6^ adversity based on the Family Adversity Index (cf. Rutter & Quinton, 1977)
